# Validity of InterVA model versus physician review of verbal autopsy for tracking tuberculosis-related mortality in Ethiopia

**DOI:** 10.1186/s12879-022-07193-w

**Published:** 2022-03-01

**Authors:** Haileleuel Bisrat, Tsegahun Manyazewal, Hussen Mohammed, Bilal Shikur, Getnet Yimer

**Affiliations:** 1 Center for Innovative Drug Development and Therapeutic Trials for Africa (CDT-Africa), College of Health Sciences, Addis Ababa University, P.O. Box 9086, Addis Ababa, Ethiopia; 2 Department of Public Health, College of Medicine and Health Science, Dire Dawa University, Dire Dawa, Ethiopia; 3 Department of Public Health, College of Health Sciences, Addis Ababa University, Addis Ababa, Ethiopia

**Keywords:** Tuberculosis, Mortality, Verbal autopsy, InterVA, Cause of death, Ethiopia

## Abstract

**Background:**

In most African countries where a legitimate vital registration system is lacking, physicians often review verbal autopsy (VA) data to determine the cause of death, while there are concerns about the routine practicality, accuracy, and reliability of this procedure. In Ethiopia where the burden of tuberculosis (TB) remains unacceptably high, reliable VA data are needed to guide intervention strategies. This study aimed to validate the InterVA model against the physician VA in tracking TB-related mortality in Ethiopia.

**Methods:**

From a sample of deaths in Addis Ababa, Ethiopia, VAs were conducted on TB-related mortality, physician-certified verbal autopsy (PCVA) through multiple steps to ascertain the causes of death. InterVA model was used to interpret the causes of death. Estimates of TB-related deaths between physician reviews and the InterVA model were compared using Cohen’s Kappa (k), Receiver-operator characteristic (ROC) curve analysis, sensitivity, and specificity to compare agreement between PCVA and InterVA.

**Results:**

A total of 8952 completed PCVA were used. The InterVA model had an optimal likelihood cut-off point sensitivity of 0.64 (95% CI: 59.0–69.0) and specificity of 0.95 (95% CI: 94.9–95.8). The area under the ROC curve was 0.79 (95% CI: 0.78–0.81). The level of agreement between physician reviews and the InterVA model to identifying TB-related mortality was moderate (k = 0.59, 95% CI: 0.57–0.61).

**Conclusion:**

The InterVA model is a viable alternative to physician review for tracking TB-related causes of death in Ethiopia. From a public health perspective, InterVA helps to analyze the underlying causes of TB-related deaths cost-effectively using routine survey data and translate to policies and strategies in resource-constrained countries.

## Background

Tuberculosis (TB) remains one of the major public health threats worldwide [1]. According to the WHO 2020 annual TB report [2], an estimated 10 million people fell ill with TB in 2019, and close to half a million people developed rifampicin-resistant TB (RR-TB), of which 78% had multidrug-resistant TB (MDR-TB). In Ethiopia, TB is still a major public health concern [3–6]. The country is among the 30 countries with the highest burden of TB, TB/HIV, and multi-drug resistant TB. Although the TB burden in Ethiopia has steadily declined, the estimated incidence remains high at 151 per 100,000 populations and a death rate of 24 per 100,000 population.

In developing countries and Africa in particular, accurate and reliable data on causes of death is essentially lacking [7–9]. Ethiopia is one of those countries with an impaired vital registration system [9–11]. Mortality estimates, so far, are derived poorly from demographic and health surveys, surveillance systems, and mathematical models. The Addis Ababa Mortality Surveillance Program (AAMSP) is a unique undertaking, which monitors citywide mortality based on burial surveillance in all cemeteries within the city boundary [12]. The program collects further information on causes of death and other socio-demographic characteristics through Verbal Autopsy (VA) from cases sampled out of burial registries.

The verbal autopsy expert algorithm (InterVA) is a compter-driven model developed and used to interpret VA data into probable causes of death [13]. It calculates the probability of a set of causes of death, given the presence of indicators (circumstances, signs, and symptoms) reported in VA interviews [14]. It is faster and cheaper, although statistical modeling of this sort may not reflect the subjective subtleties of physicians’ review [15], it is advantageous in terms of efficiency, consistency, and standardization. As a result, there is an increasing trend to shift from physician’s based review to InterVA model; however, the reliability of this model across different settings has not been studied well [15].

In most of the African countries, including Ethiopia, where a legitimate vital registration system is lacking, physicians often review VA data to determine the cause of death, while there are concerns on the routine practicality, accuracy, and reliability of this procedure. In Ethiopia where the burden of TB remains unacceptably high, reliable VA data are needed to guide decision-making strategies. The country needs reliable data on the level and causes of TB-related mortality for effective TB program implementation and realization of the End TB strategy by 2035. This study aimed to validate the InterVA model against the physician VA in tracking TB-related mortality in Ethiopia.

## Method

This study used a completed verbal autopsy between 2007 and 2017. The cause of death assigned by physicians and InterVA was compared at the individual level.

### Source of data

The source of data was from VA and physician review records of the AAMSP database. The AAMSP used to register all deaths that happen in Addis Ababa on regular basis. The primary source of AAMSP data was death registration followed by verbal autopsy interviews. Information gathered from a standardized interview about the circumstances of death from relatives or friends or close caregivers of the deceased was reviewed by physicians to assign causes of death. The AAMSP was established in 2001 in Addis Ababa, the capital of Ethiopia with the main objective of monitoring cause-specific mortality with a special focus on HIV/AIDS. From a total of 89 cemeteries, the program collected information about decedents' background information and lay the cause of death from family members[13, 16].

### Data collection

#### Burial surveillance

A burial surveillance form has been used by cemetery clerks who trained about death registrations in training workshops. In each of the cemeteries, one or two cemetery-based clerks were assigned to register deaths using the burial surveillance form prepared for this purpose. The variables registered by the cemetery clerks include the name of the deceased, date of burial, age, sex, birth region, marital status, ethnicity, religion, specific address, and lay cause of death. More than 15,000 deaths have been registered annually from all the cemeteries. The data that was collected from all cemeteries of Addis Ababa was entered into computer software to serve as a sampling frame for VA.

#### Verbal autopsy

A random sample of about ten percent of burial records from all cemeteries except ‘Baytewar’ cemetery was selected for VA interviews. ‘Baytewar’ is an Amharic word used to refer to a stranger or someone who is socially isolated. In Baytewar cemetery, bodies with no close relatives or friends to facilitate a funeral were buried. The Baytewar cemetery alone accommodated around 15% of the total number of burials. Most of these were infant bodies delivered by the obstetric wards of hospitals and remain unidentified cemetery and those with complete addresses were not eligible for VA interviews.

Verbal autopsies were conducted by trained interviewers after 2 to 3 months of the mourning period. The interviewers were trained on how to contact respondents when to interview and complete the questionnaires. Of the total records that were sampled for VA interviews, about 7.6% were not completed with the main reasons either respondents refused the interview or the households were not found.

VA employs a standardized questionnaire to produce information about the causes of death. Through this process, the information on the sign, symptoms, medical history, and circumstances preceding deaths were elicited by interviewing the next kin or caregiver. The cause or sequence of causes that led to death were assigned based on data collected from the questionnaire and any health records and narrative section that has been available. The VA questionnaire was adapted from one used at the INDEPTH Network site [17] and included a set of questions previously used in Ethiopia [18–20] and modified on the WHO 2012 VA instrument developed by the WHO, Health Metrics Network (HMN), and the IN-DEPTH-Network [21].

#### Physician review

Physicians reviewed the completed VA questionnaire to ascertain the causes of deaths which were done in multiple steps. Initially, the cause of death was assigned independently by two physicians after they review the completed VA questionnaire. Then, the two-physicians diagnosis was checked by the surveillance team members. If the two-physician diagnosis (for the assigned cause of death) contradicts, a third physician reviewed the case, and the final assignment was made based on the agreement between any two of the three physicians. However, if the assigned cause of death by the third physician was not in agreement with any of the two-physician diagnoses, the cause for death was assigned on a panel discussion between them. In the situation where the three physicians could not reach an agreement, the cause of death was assigned as undetermined. The physicians were participating to review the process which was second- or third-year internal medicine residents of Addis Ababa University recruited to join the university after serving two or more years as a General Practitioner (GP) in any of the public hospitals. We provided them training and annual refreshments on the standard verbal autopsy method.

#### InterVA

The physician and the InterVA-3.2 model independently assessed the same basic data from the VA questionnaire. The study tested the concordance of assigning any kind of TB as a cause of death between physician review and the interval model. The model's input data include signs, symptoms, medical history, and situations collected from the VA questionnaires' close-ended questions. Compiling the same VA data into an input file for the InterVA model and processing it into the cause of death data, data adaptations were made to match the model. The model additionally demands a "high" or "low" input to describe the local prevalence of two specific causes, which can vary by order of magnitude between settings. The WHO verbal autopsy tool does not have data on some InterVA markers, thus they were left blank.

A STATA do file used to validate the model in 2003 was revised and applied to produce parameters essential for the model. Kappa statistics, ROC curve, sensitivity, and specificity were applied to compare agreement between PCVA and InterVA. Causes of death are assigned to a predefined matrix of evaluated probabilities of occurrence.

#### Interpretation of the InterVA model

The model relates a range of input indicators, like sex, age, physical signs and symptoms, medical record, and therefore the circumstances of death to likely CODs using Bayesian probabilities [22]. The model leads to up to 3 likely causes per case when possible; each related to a quantified likelihood. To assign an estimate of the certainty for that patient, the model gives the common likelihood for a maximum of three CODs [23]. during this study, a high prevalence of Malaria and HIV/AIDS were used as basic epidemiological parameters for the model as their prevalence varies from place to put. Data were entered case-by-case into Microsoft visual FoxPro window of the InterVA version 3.2 to assign the possible COD responsible for the death of every individual.

### Distribution of N subjects by physician review and InterVA model category

The formula we used to determine the distribution of subjects by physician review and InterVA model category was:

$${\text{K}} = \frac{{{\text{P}}_{{\text{o}}} - {\text{P}}_{{\text{e}}} }}{{1 - {\text{P}}_{{\text{e}}} }} = \frac{{1 - 1 - {\text{P}}_{{\text{o}}} }}{{1 - {\text{P}}_{{\text{e}}} }}$$

where P_o_ = the relative observed agreement among raters.

P_e_ = the hypothetical probability of chance agreement

K = Kappa statis

**Table:** 

		Physician review			
		Category 1 (Yes)	Category 2 (No)	Total	
InterVA Model	Category 1 (Yes)	a	B	a + b	P_1_. = (a + b)/N
	Category 2 (No)	c	D	c + d	P_2_. = (c + d)/N
	Total	a + c	b + d	N	
		P 0.1 = (a + c)/N	P 0.2 = (b + d)/N

$$Po=\frac{a+d}{N}$$

where a = the total number of instances that both raters said were correct. The raters are in agreement.

b = the total number of instances that rater 2 said was incorrect, but rater 1 said were correct. This is also disagreement.

c = the total number of instances that rater 1 said was incorrect, but rater 2 said were correct. This is also disagreement.

d = the total number of instances that both raters said was incorrect. Raters are in agreement.

In Cohen’s kappa, the chance agreement is defined as the sum of the products of marginal distributions, i.e.

$$Pe\left( k \right)\, = \,P.{1}*P{1}.\, + \,P.{2}*P{2}{\text{.}}$$

### Receiver operator characteristics (ROC) curve

For both PCVA and InterVA, the area under the receiver operator characteristics (ROC) curve was used to assess overall diagnostic performance (properly diagnosing all diseases). The area under the curve (AUC) of a procedure should be near one for it to be highly sensitive and specific. The approach is more accurate if the curve closely aligns the left-hand border and the top border of the ROC space. If the area under the ROC curve was more than 0.75, we evaluated our methods to be appropriate.

### Validity measures: sensitivity and specificity

Sensitivity and specificity with their 95% confidence intercal (CI) were compared for PCVA and InterVA model. The formula for the calculation were defined as:

$${\text{Sensitivity}} = \frac{{{\text{TP}}}}{{({\text{TP}} + {\text{FN}})}}$$

$${\text{Specificity}} = \frac{{{\text{TN}}}}{{({\text{FP}} + {\text{TN}})}}$$

Where: TP = true positive; FP = false positive; TN = true negative; FN = false negative.

### Inclusion and exclusion criteria

The inclusion criteria were all records where causes of death were assigned and those with all the information like identification of decedents, sex, age, house address, and date of birth.

The exclusion criteria were records with missing in address, name, inconsistency in the address including place of burial. Causes of death assigned by the InterVA model as “Indeterminate” were also excluded from the analysis.

### Data management and analysis

Data analysis was conducted using STATA 14 software. Individual decedents had a unique ID and all datasets were merged using this ID before analysis. Two separate variables (TB_inerva and TB_pr) were generated for comparison purposes and documenting the trend of TB-related death. Coding and recoding, labeling, and analysis were done.

Estimates of TB-related deaths between physician reviews and the InterVA model were compared using Cohen’s Kappa (k) and ROC curve analysis. A Kappa value of < 0 indicates no agreement and 0–0.20 as slight, 0.21–0.40 as fair, 0.41–0.60 as moderate, 0.61–0.80 as substantial, and 0.81–1 as almost perfect agreement.

In this paper, sensitivity refers to the ability of the InterVA model to correctly identify TB-related deaths as assigned by the physician method; whereas specificity refers to the proportion of Death from other causes that are correctly identified as non TB. These two measures were closely related to type I and type II errors. Both sensitivity and specificity were calculated.

### Ethical considerations

The study has been reviewed and approved by the Ethiopian National Research Ethics Review Committee of the Ethiopian Ministry of Science and Technology, and the Institutional Review Board of the College of Health Sciences, Addis Ababa University. Informed consent was obtained from caregivers or another eligible adult in the family during the VA interview. Access to the AAMSP data was obtained from the Addis Ababa University Mortality surveillance team that manages the project. The participants remained anonymous and were not identified in the research report or in other means used to disseminate findings.

## Results

### Background characteristics

A total of 8952 VA was completed with physician diagnosis in the period September 2007 to 2017. Of these, 4618 (51.6%) were male and 4086 (45.6%) were from the age group 65 years and above. Regarding educational status, 3836 (42.8%) had completed elementary and/or secondary school, while 3721 (41.6%) were illiterates. About 3936 (44%) and 2471 (27.6%) of decedents were married and widowed, respectively (Table 1).

**Table 1:** Background characteristics of decedents 2007–2017 Addis Ababa, Ethiopia

Background characteristics	Number	Percent
Sex		
Male	4618	51.59
Female	4334	48.41
Age		
15–24	382	4.27
25–34	1019	11.38
35–44	1124	12.56
45–54	1071	11.96
55–64	1270	14.19
65+	4086	45.64
Education		
No formal school	3721	41.6
Primary	2025	22.6
Secondary	1811	20.2
Tertiary +	833	9.3
Unknown	303	3.4
Marital status		
Single	1648	18.41
Married	3936	43.97
Separated/divorced	852	9.3
Widowed	2471	27.60
Unknown	45	0.50

### TB-related cause of death

From the total 8952 PCVA conducted, 972 (10.9%) were assigned as TB-related deaths by physicians. Of these, 313 VA were excluded from the InterVA analysis due to important missing variables. Of the 8,639 VAs able to be analyzed using InterVA, 975 (11.3%) were assigned as TB-related deaths (Table 2).

**Table 2:** Comparison of all causes of death by Physician and InterVA model

			InterVA model	
Year	Physician review	Cause of death with non-TB	Cause of death with TB	Cause of death with non-TB
2007		874 (76.5)	221 (20.1)	875 (79.8)
2008	224 (19.9)	902 (80.1)	184 (16.9)	908 (87.5)
2009	136 (11.9)	1009 (88.1)	138 (12.5)	966 (12.7)
2010	98 (9.2)	966 (90.8)	102 (9.9)	930 (90.1)
2011	86 (8.1)	978 (91.9)	96 (9.3)	934 (90.7)
2012	101 (9.3)	984 (90.7)	114 (10.9)	931 (89.1)
2015	26 (3.8)	664 (96.2)	51 (7.7)	612 (92.3)
2016	20 (2.3)	840 (97.7)	45 (5.5)	778 (94.4)
2017	13 (1.7)	763 (98.3)	24 (3.2)	730 (96.8)
Total	972 (10.9)	7980 (89.1)	975 (11.3)	7664 (88.7)

**Fig. 1:**
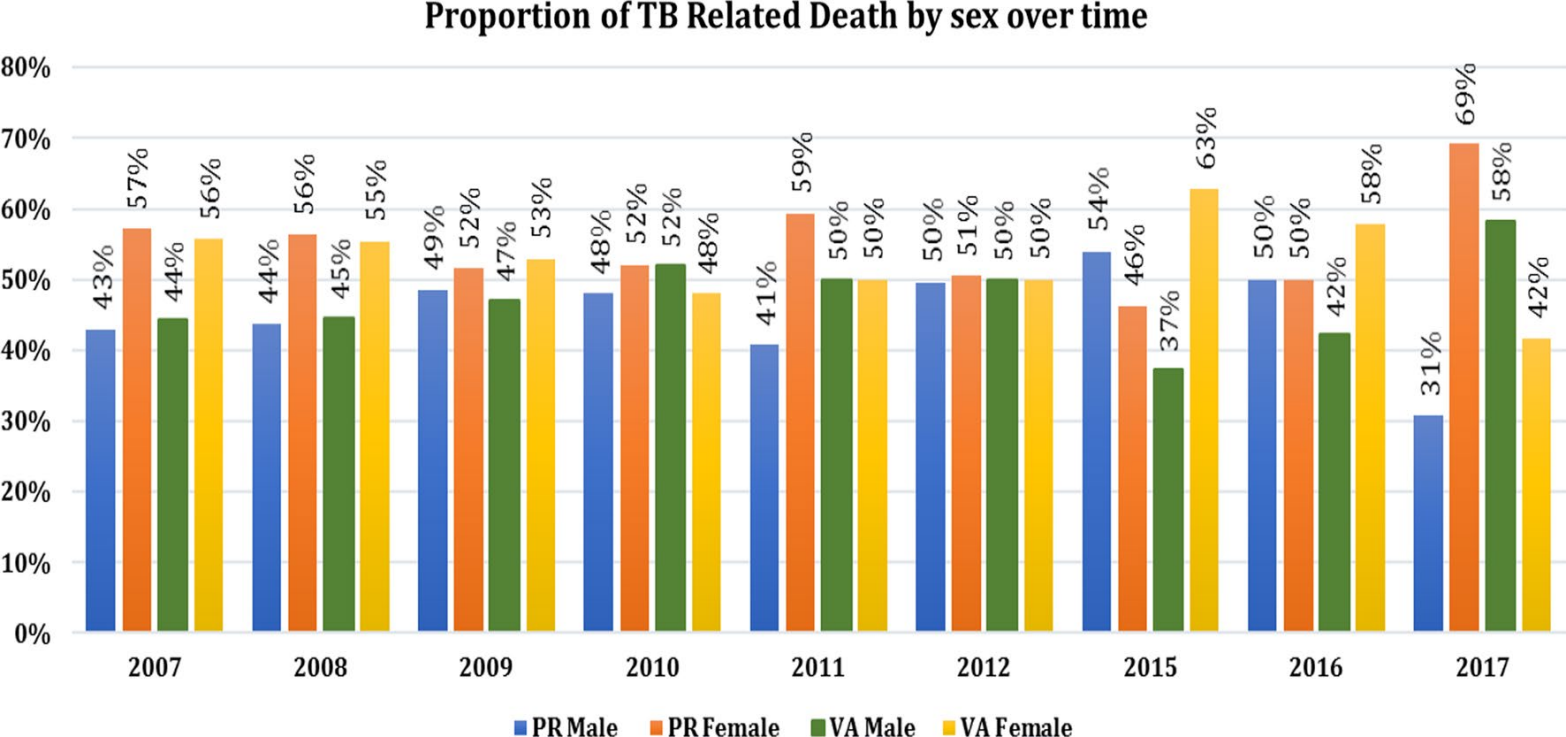
Proportion of TB-related deaths based on gender in physician review and InterVA model in the years 2007 to 2017

Of those classified by physicians as TB-deaths, 533 (54.8%) were female, and from the InterVA model, 520 (53.3%) were female. The proportion of male TB-related death by Inter VA model was comparable with physician review, 65 (47.1%) and 66 (48.5%), for the period 2009. In the same period, the proportion of female TB-related death by InterVA and physician review were 73 (52.9%) and 70 (51.5%), respectively. However, male and female TB-related death varies from year to year for both InterVA model and physician review (Fig. 1).

### Agreement in diagnosis between physician and InterVA Model

According to the analysis, the results of the level of agreement was 92.9%, with a kappa value of 0.59 (95% CI: 0.57–0.61), indicating that there is a moderate agreement (Table 3).

**Table 3:** Distribution of N subjects by physician review and InterVA model category

		Physician review		
		TB-related death	Death from other cause	Total
InterVA Model	TB-related death	622 (a)	353 (b)	975
	Death from other cause	350 (c)	7314 (d)	7664
	Total	972	7667	8639 (N)

The observed agreement was defined as:

$$Po=\frac{622+7314}{8639}=0.918$$

$${\text{Pe}}\left( {\text{k}} \right) = \left( {0.{112}*0.{112}} \right) + \left( {0.{887}*0.{887}} \right)$$

$$Pe\left( k \right) = 0.{799}$$

$$\mathrm{k}=\frac{0.918-0.799}{(1-0.799)}= 0.59$$

From this analysis, the level of agreement in the Kappa value was 0.59, which indicates that there was a moderate agreement.

### ROC analysis

**Fig. 2:**
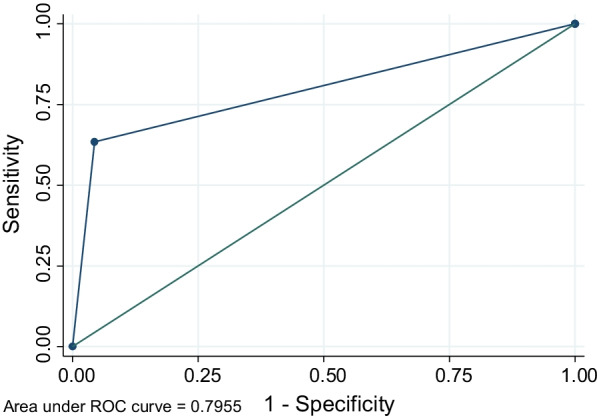
Receiver operating characteristic curve of sensitivity and specificity

Figure 2 presented the ROC analysis in a one-to-one square. The area under the curve captures the relationship between the sensitivity and specificity of the Inter VA method and is indicative of how the method performed. The curve follows the left-hand border and then the top border of the ROC space indicating an acceptable level of accuracy.

In this study, the ROC curve indicated that the InterVA model predicted the cause of TB-related death with an area under curve or probability of 0.79 when compared with physician review which indicates the good diagnostic performance of the method. The sensitivity and specificity of the InterVA model were 0.64 and 0.95, respectively.

## Discussion

This study compared the cause of TB-related death agreement between the InterVA model and physician review methods. The study showed that level of agreement between physician review and the InterVA model was 92.9% with the kappa value of 0.59, which indicated a moderate agreement. The finding was similar to a previous study in the country [24], Kappa 0.58% (95% CI: 0.50–0.65), but higher when compared with another study[25], kappa = 0.50, (95% CI: 0.4–0.6). This indicated the potential of the IntraVA model to be used to establish TB-related death data.

When it comes to assigning cause-specific mortalities using VA data at the population level, the probabilistic InterVA model produced substantially similar results as the physicians in this study, which was in line with previous studies [14, 26]. The InterVA model's cause-specific mortality data was consistent with existing knowledge about the burden of diseases in the sub-Saharan Africa context [27, 28], suggesting that the model performed well in generating cause-specific mortality data from VA.

The study finding showed that the overall cause of TB-related death both by physicians and the model were low when compared with previous studies from Ethiopia which has given a kappa value of 31%, with TB-related death to be 36.0% and 23.0% by the InterVA model and the physicians respectively [24]. This variation might be due to the study population and time of the study.

The issue of how to obtain a true gold standard in VA validation research arises repeatedly. CODs based on hospital diagnoses have been considered the gold standard in many studies [29, 30]. Hospital diagnoses, on the other hand, have limitations as a gold standard since the composition and distribution of hospital CODs may not be indicative of community mortality. Furthermore, in resource-constrained healthcare settings, when hospital diagnoses are available, they are of poor quality and are limited by insufficient clinical data and record keeping [31–33]. Moreover, hospital users and residential users may have varied abilities to notice, recall, and report indicators of sickness. Physician review was used as a reference standard in this study to investigate InterVA. For this study's population, physician review was the only option for COD assessment. This option, however, has drawbacks. Physicians' experiences, perceptions, and interpretations of local epidemiology might contribute to variations in COD data, making it difficult to make valid temporal and regional comparisons. Furthermore, they frequently make decisions based on open history and may not account for all indicators consistently. Our report showed that the use of the physician review has helped to find the most relevant facts relating to the cause of death by tuberculosis, but the choice had limitations. The physicians have considered the detailed information by going through the questionnaire, using their clinical skills and experiences in determining the cause of death. They might however be influenced by their own biases.

Deaths during TB treatment signify gaps in the accurate implementation of TB programs [34, 35] and this has been higher in countries like Ethiopia where the healthcare system is hindered by infrastructure and human resource constraints [36–39]. The current study sheds light on the role and feasibility of using the InterVA model alternative to physician review of verbal autopsy that competes for the scarce health human resources.

The limitation of this study was that it used secondary data for the primary analysis which is because the questionnaire is developed for all causes of death not specifically for TB-related death identification. Another drawback of using physician review as a gold standard is that physicians may misinterpret some of the VA data, leading to a potentially incorrect cause of death result. The experience, observation, and interpretation of the physicians might also influence interpretations and reach a biased decision of cause of death. Otherwise, the study was conducted carefully and the data management and analysis were conducted in line with the standard and appropriate procedures and statistics methods.

## Conclusion

The InterVA model is a viable alternative to physician review for tracking TB-related causes of death in Ethiopia. From a public health perspective, InterVA helps to analyze the underlying causes of TB-related deaths cost-effectively using routine survey data and translate to policies and strategies in resource-constrained countries.

## Data Availability

The dataset supporting the conclusions of this article is included in the article.

## Abbreviations

VA: Verbal autopsy
TB: Tuberculosis
PCVA: Physician-certified verbal autopsy
k: Cohen’s Kappa
MDR-TB: Multidrug-resistant TB
RR-TB: Rifampicin-resistant TB
AAMSP: Ababa Mortality Surveillance Program
GP: General practitioner
PR: Physicians review
WHO: World Health Organization
ROC: Receiver operator characteristics
InterVA: Verbal Autopsy Expert Algorithm
